# Highly specific and label-free histological identification of microcrystals in fresh human gout tissues with stimulated Raman scattering

**DOI:** 10.7150/thno.53755

**Published:** 2021-01-01

**Authors:** Bohan Zhang, Hanlin Xu, Jun Chen, Xiaoxia Zhu, Yu Xue, Yifan Yang, Jianpeng Ao, Yinghui Hua, Minbiao Ji

**Affiliations:** 1 Department of Sports Medicine, Huashan Hospital, Fudan University, Shanghai 200040, China.; 2 State Key Laboratory of Surface Physics and Department of Physics, Human Phenome Institute, Multiscale Research Institute of Complex Systems, Academy for Engineering and Technology, Key Laboratory of Micro and Nano Photonic Structures (Ministry of Education), Fudan University, Shanghai 200433, China.; 3 Department of Rheumatology, Huashan Hospital, Fudan University, Shanghai 200040, China.

**Keywords:** gout, stimulated Raman scattering, monosodium urate, label-free histology

## Abstract

Gout is a common metabolic disease with growing burden, caused by monosodium urate (MSU) microcrystal deposition. In situ and chemical-specific histological identification of MSU is crucial in the diagnosis and management of gout, yet it remains inaccessible for current histological methods.

**Methods:** Stimulated Raman scattering (SRS) microscopy was utilized to image MSU based on its fingerprint Raman spectra. We first tested SRS for the diagnosis capability of gout and the differentiation power from pseudogout with rat models of acute gout arthritis, calcium pyrophosphate deposition disease (CPDD) and comorbidity. Then, human synovial fluid and surgical specimens (n=120) were were imaged with SRS to obtain the histopathology of MSU and collagen fibers. Finally, quantitative SRS analysis was performed in gout tissue of different physiological phases (n=120) to correlate with traditional histopathology including H&E and immunohistochemistry staining.

**Results:** We demonstrated that SRS is capable of early diagnosis of gout, rapid detection of MSU in synovial fluid and fresh unprocessed surgical tissues, and accurate differentiation of gout from pseudogout in various pathophysiological conditions. Furthermore, quantitative SRS analysis revealed the optical characteristics of MSU deposition at different pathophysiological stages, which were found to matched well with corresponding immunofluorescence histochemistry features.

**Conclusion:** Our work demonstrated the potential of SRS microscopy for rapid intraoperative diagnosis of gout and may facilitate future fundamental researches of MSU-based diseases.

## Introduction

Gout is a crystal-deposition disease caused by uric acid disturbance, and has attracted growing interests of clinicians and researchers due to its rising prevalence and disease burden 1. DALYs (disability adjusted life year) of gout has increased by 50% in the past 20 years 2. MSU crystalizes and deposits on joint surfaces, leading to persistent inflammation and damage of joint structures, which in turn favors MSU invasion and further accelerates the course of gouty arthritis (GA) 3. Moreover, MSU deposition may also increase the risk for cardiovascular and renal disease and impairs quality of life 4.

The first-line strategy for the management of chronic gout is to reduce MSU deposition by medication or surgeries 5, hence quick and accurate detection of MSU in situ is critical in both the diagnosis and treatment of gout. However, neither the gold standard histological examinations, nor compensated polarizing light microscopy (CPLM) could provide sufficient chemical specificity of MSU crystals 6. Chemical specific detection is particularly important in complex situations including the differential diagnosis from pseudogout, the occurrence of MSU deposition in the asymptomatic population, and the increased number of comorbidity cases 2. Both CPLM and H&E (Hematoxylin & eosin) bear relatively high false negative rates, and suffer from time-consuming tissue processing and the requirement of experienced pathologists 7.

Although crystal identifications were performed mostly in synovial fluid 8, it is worth noting that gout population may experience atypical disease processes. MSU crystal deposition were found on the joint surface of many patients before their first onset 7. A considerable number of patients with gout often delay diagnosis till the first symptom of osteochondral injury, ligament injury, and even fracture 9, 10. Moreover, gout arthritis can be confusing among suspected infection, tumor, or rheumatoid arthritis, until postoperative pathology was conducted 11. Therefore, intraoperative diagnostic methods capable of rapid and specific identification of MSU in fresh tissues are highly desired for the optimization of operative strategy 12.

Raman scattering spectroscopy provides intrinsic vibrational fingerprints of chemical bonds for specific detection and analysis of biomolecules. Although spontaneous Raman spectroscopy of MSU in gout and calcium pyrophosphate dihydrate (CPPD) in pseudogout have been investigated in previous studies 13, the weak signal intensity has prevented its application for rapid histology. Stimulated Raman scattering (SRS) microscopy is a relatively new technique with ~10^3^-10^5^ enhanced Raman signal due to the coherent stimulated emission process 14, enabling high-speed imaging up to video rate (~25 frames/s) 15. Meanwhile, SRS microscopy inherits the spectroscopic advantages of spontaneous Raman, and has been developed as a fast and label-free imaging technique with high sensitivity and chemical specificity 16-19, showing potentials in many branches of biomedical researches, including cell biology, lipid metabolism, microbiology, tumor detection, protein misfolding, and pharmaceuticals^20^-^24^. Particularly, SRS has shown success in rapid histopathology for fresh surgical tissues and intraoperative diagnosis, yielding near-perfect agreements with conventional H&E staining 25, 26. In addition, SRS is capable of performing quantitative chemical analysis for mixtures of multiple components, based on the spectral information of each species 19, 20, 27.

In this report, we demonstrated the feasibility of SRS to specifically image MSU depositions in gout tissues in situ based on its fingerprint Raman peaks. Simultaneous imaging of MSU and collagen fibers has been realized with the combination of SRS and second harmonic generation (SHG) microscopy. By evaluating various MSU depositions in both rat model and human surgical specimens, we were able to show the early diagnosis of gout, and differentiation of gout from pseudogout based on the distinct Raman features of MSU and CPPD. Furthermore, comparing with H&E and immunohistochemical staining, our quantitative and statistically results showed that MSU contents moderately correlate with the expression level of inflammatory cytokines, which was consistent with the previous studies on the structure of tophi and neutrophil extracellular traps (NETs) 28. These results indicate that SRS microscopy may provide ideal analytical tool for learning the fundamental interactions between MSU and the surrounding tissues, and hold potential for rapid clinical diagnosis of gout and other MSU-based diseases.

## Results

### Raman spectral characterization of MSU

**Figure 1:**
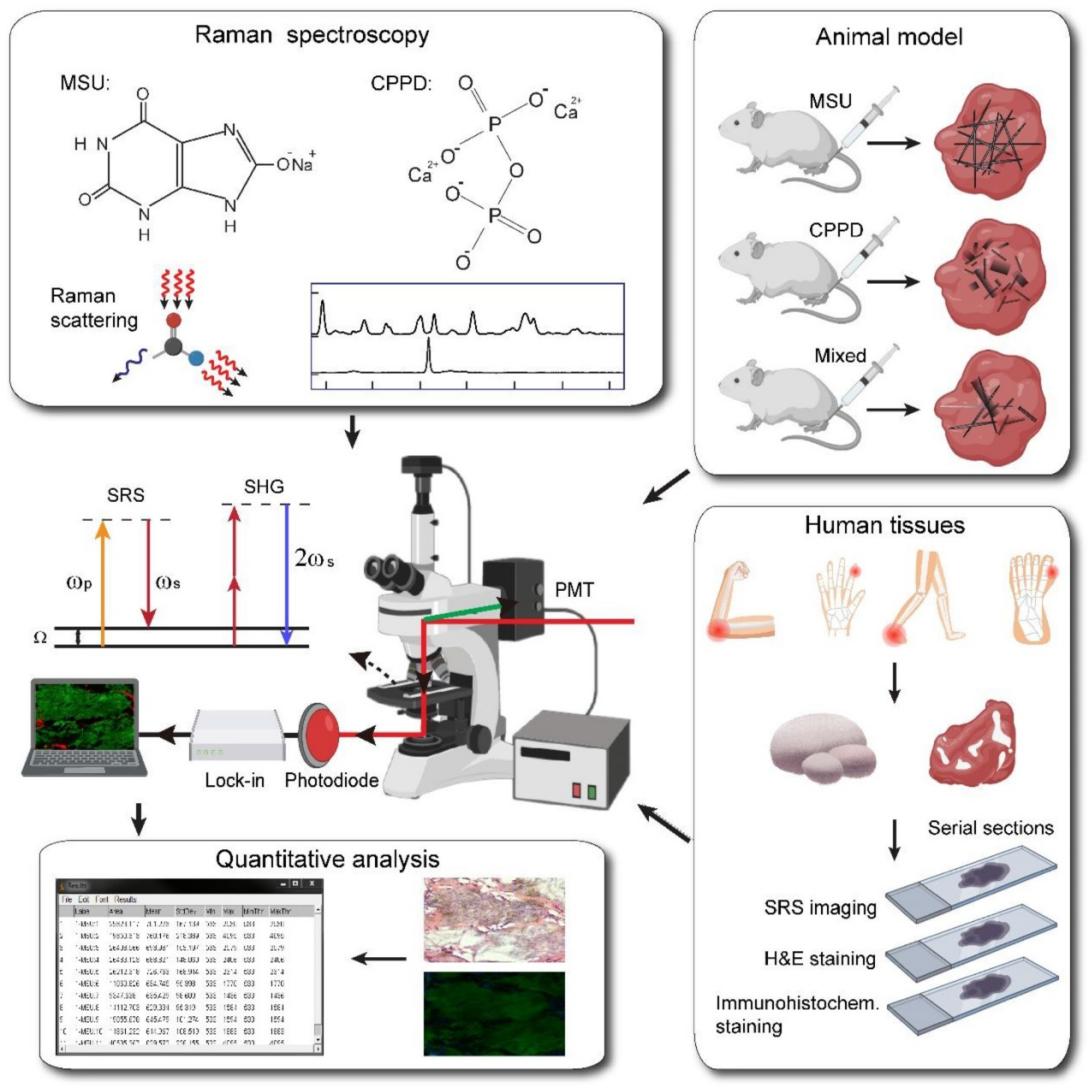
**Schematics of the experimental design.** Left top, spontaneous Raman characterization; Right top, acute GA rat models of MSU, CPDD and comorbidity for testing the capabilities of early diagnosis of gout and differentiation power from pseudogout; Right bottom, human surgical specimens were harvested from different locations; All specimens were imaged with SRS/SHG microscopy to obtain the distributions of MSU and collagen fibers; Left bottom, quantitative SRS analysis was performed to correlate with traditional histopathology including H&E and immunohistochemistry staining.

**Figure 2:**
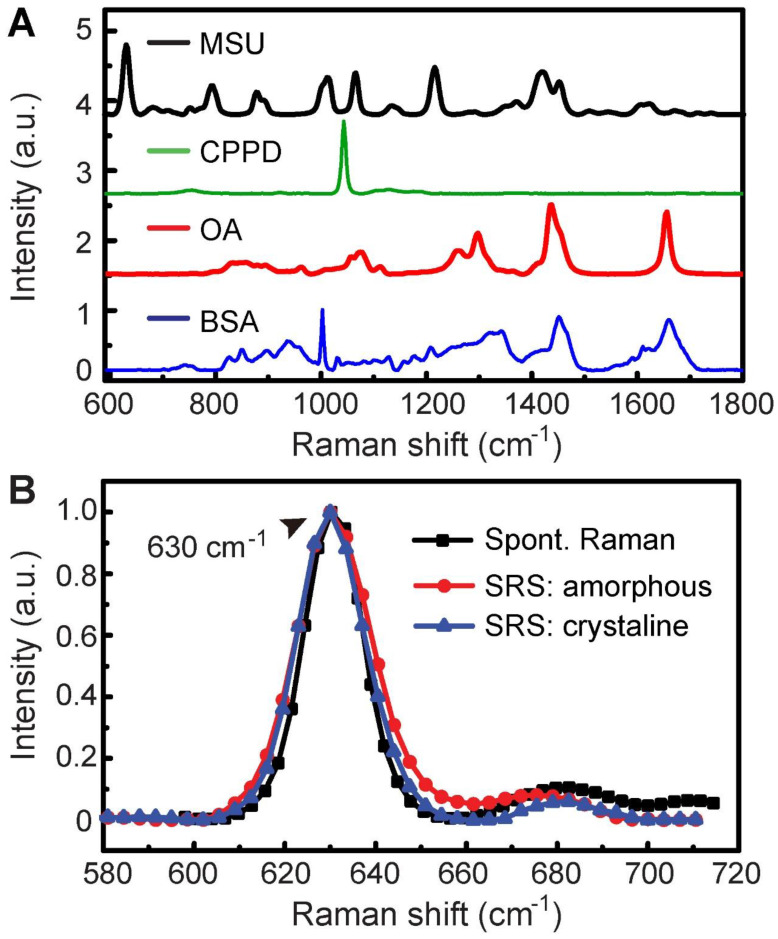
**Spontaneous and stimulated Raman spectra of standard chemicals. (A)** Spontaneous Raman spectra of MSU, CPPD, lipid (OA) and protein (BSA). **(B)** Raman and SRS spectra of crystalline and amorphous MSU samples.

The overall experimental design is illustrated in Figure 1, including the processes of taking Raman spectroscopy, constructing rat models with MSU and CPPD crystals, harvesting human surgical specimens, followed by imaging with SRS/SHG microscopy, and finally performing data analysis. We first characterized the spontaneous Raman spectra of standard MSU, CPPD, protein (bovine serum albumin, BSA) and lipid (oleic acid, OA), showing a variety of Raman peaks/bands representing the characteristic features of each chemical (Figure 2A and Figure S1). Among these Raman peaks, the one at 630 cm^-1^ was identified as the spectral “marker” for MSU, not only because it has the highest intensity, but also for its clean background, i.e. no background spectra from the tissue, purine or pyrimidine derivatives would effectively interfere with the MSU spectra (Figure 2A and Figure S2). This low-frequency Raman peak could be assigned to the purine ring breathing vibrational mode of MSU molecule 29. Other Raman peaks of MSU could also be identified and used for MSU imaging, such as the ones at 1011 cm^-1^ and 1066 cm^-1^ (Figure 2A), as will be presented later. Both the spontaneous and stimulated Raman spectra of MSU samples were measured and found to agree very well with each other (Figure 2B), which provides the basis for SRS imaging of MSU throughout the work. It is worth mentioning that such a low-frequency Raman mode is usually difficult to detect with coherent anti-Stokes Raman (CARS) microscopy, in contrast to the much more feasible measurement with SRS.

### SRS/SHG imaging of crystalline and amorphous MSU

**Figure 3:**
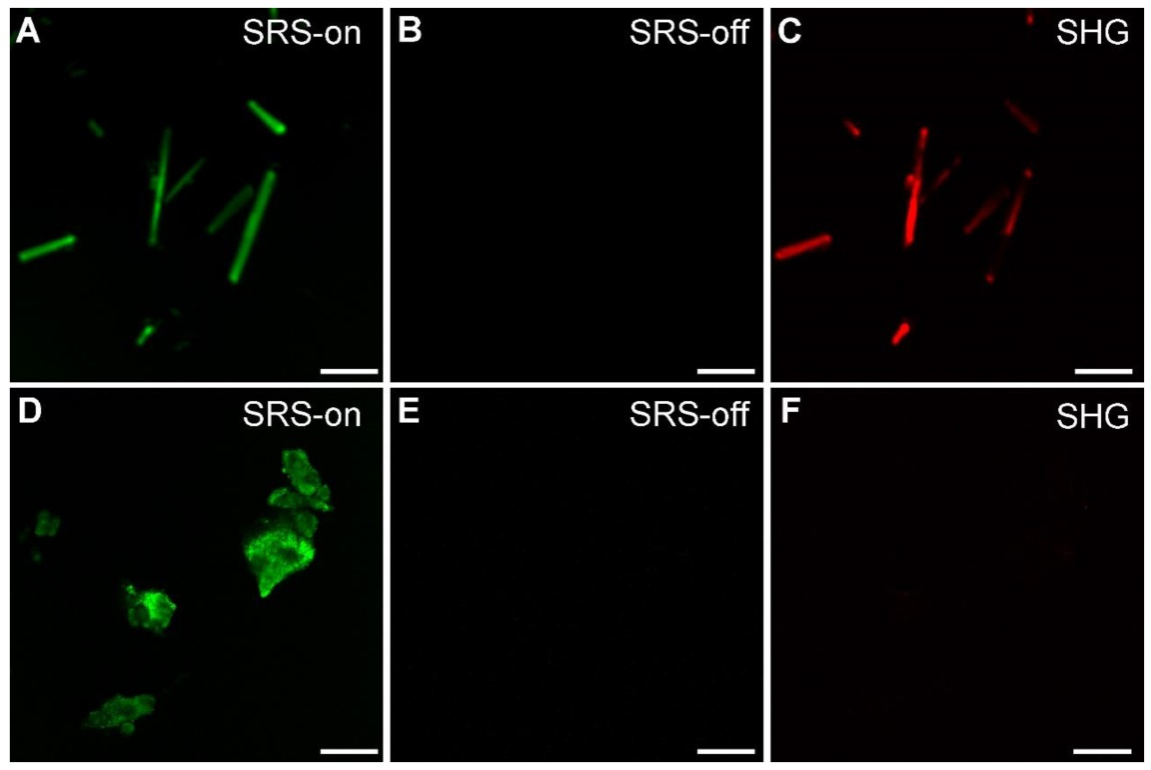
**Imaging and differentiation of crystalline and amorphous MSU with SRS and SHG microscopy. (A)** on-resonance SRS image of MSU crystals at 630 cm^-1^; **(B)** off-resonance SRS image takend at 700 cm^-1^; (C) SHG image of the same crystals; (D-F) corresponding SRS and SHG images of amorphous MSU. Scale bar: 5 μm.

We applied SRS and SHG microscopy to simultaneously characterize MSU in both crystalline and amorphous forms. The optical transition diagrams of SRS/SHG are illustrated in Figure 1, and the microscope setup could be found in Figure S3, with detailed description of the system shown in Methods and previous publications 16. Under bright field microscope, MSU crystals appear typical needle-like shapes (Figure S4). These crystals could be readily imaged with SRS based on the 630 cm^-1^ vibration (Figure 3A), with high chemical specificity shown as the off-resonance behavior when the SRS frequency was slightly detuned away from vibrational resonance (Figure 3B). SHG is known to be sensitive to non-central symmetric structures, including MSU crystal (Figure 3C) and collagen fibers in tissues 30. However, we found linearly polarized light beams tend to generate strong anisotropic signals of both SRS and SHG with respect to the crystal orientation (Figure S5), because of the dependence of Raman polarizability tensor and second-order optical susceptibility on crystal symmetry 30. We thus applied circular polarization for both pump and Stokes beams to eliminate the orientational effect of both MSU crystals and collagen fibers. While SRS and SHG showed almost identical images of MSU crystals as in bright field microscope, careful examination revealed that SRS agrees better with the crystal morphology, whereas SHG might experience signal loss due to crystal defects and structural changes (Figure S5). More importantly, SRS has the large advantage of chemical specific imaging, regardless of crystal symmetry. As shown in Figure 3D-F, even though amorphous MSU does not generate SHG signal, it could be imaged with SRS equally well as the crystalline form, since both forms of MSU share almost identical SRS spectra around 630 cm^-1^ (Figure 2B). Therefore, the combination of SRS and SHG microscope could well-distinguish between crystalline and amorphous forms of MSU and will be applied to analyze crystallization ratio in gout tissues.

### Early detection of MSU deposition and differentiation from CPPD in acute rat models

We then verified the hypothesis that SRS could differentiate between gout and pseudogout, both of which are joint crystal-related diseases. Similar to MSU crystal being the cause of gouty arthritis, CPPD crystallization was considered to be the cause of pseudogout, hence differentiation of the two chemicals becomes the key to solve the problem. Since both CPPD and MSU crystals could generate similar intensities of SHG signal, SHG alone is incapable of distinguishing them (Figure S6). Comparing the Raman spectra of MSU and CPPD revealed that the 1050 cm^-1^ peak (P-O stretching mode) of CPPD could be used to differentiate from MSU by either the 630 cm^-1^ peak or the nearby peaks at 1011 cm^-1^ and 1066 cm^-1^ (C-C stretching vibrational mode) (Figure S6A) 29, 31. Therefore, these spectral signatures are expected to distinguish CPPD and MSU with multi-color SRS. To assess the ability of such differential imaging, SRS microscopy was applied to image mixed CPPD and MSU crystals (in 1:1 ratio) at 1050cm^-1^, 1011cm^-1^ and 1066cm^-1^, and successfully identified both chemicals within the same field of view (Figure S6).

**Figure 4:**
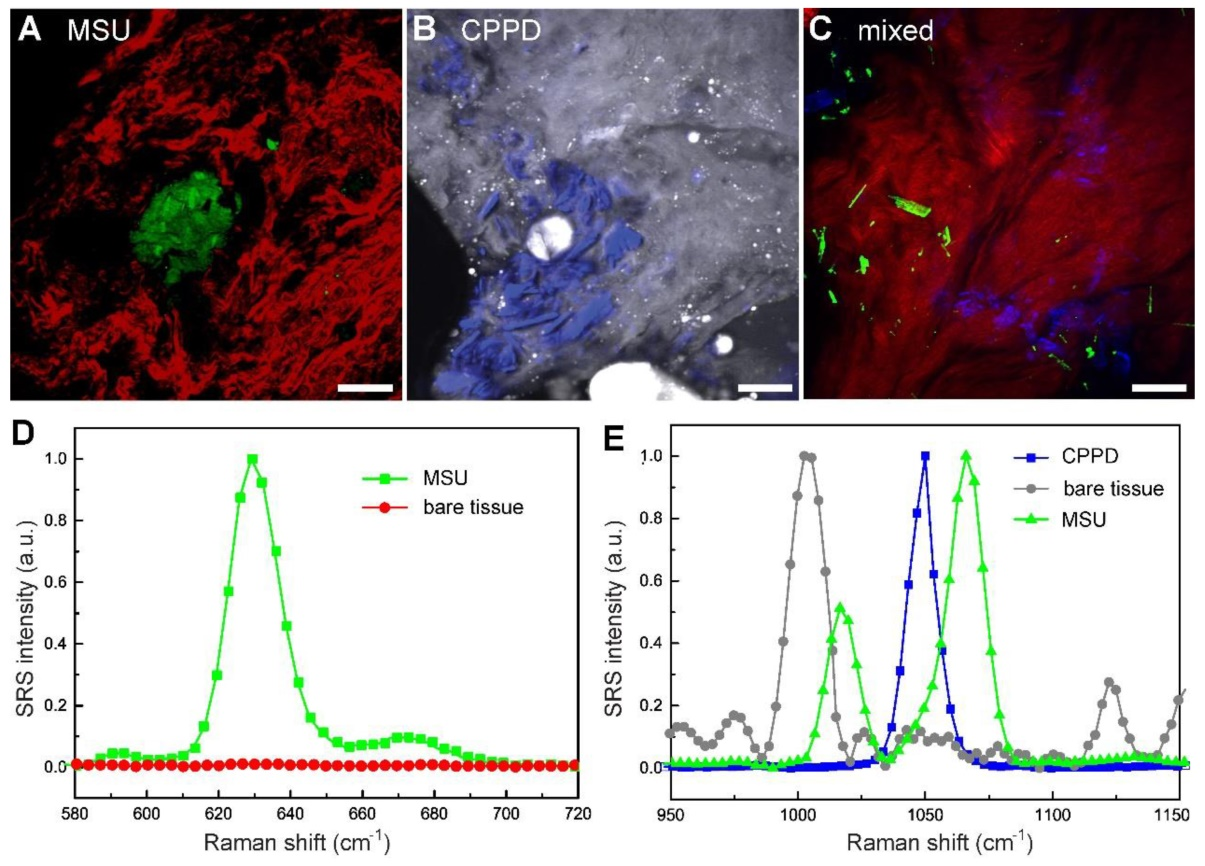
** Differentiation of pseudogout and early detection of microcrystals in fresh tissues of rat models (3 days after injection) with multicolor SRS. (A)** Depositions of MSU (green, 630 cm^-1^) surrounded by collagen (red, SHG) were shown in acute gout model (*n =* 3 rats). **(B)** Depositions of CPPD (blue, 1050 cm^-1^) were detected in the synovium protein (gray, 2930 cm^-1^) of CPPD model (*n =* 3 rats). **(C)** MSU and CPPD microcrystals were clearly distingruished in synovium tissues of combined model (*n =* 3 rats). **(D-E)** SRS spectra of MSU, bare tissue and CPPD. Scale bar: 20 μm.

To test the capability of SRS in imaging tissues, rat models of acute gout, CPDD and comorbidity were established. Rats were sacrificed 3 days after injection and fresh tissues from synovium were collected and imaged without further processing. Trace amount of MSU microcrystals could be seen using the 630 cm^-1^ mode in the tissues of gout rats (Figure 4A), which is usually difficult to detect in acute articular animal models 32. Similarly, CPPD microcrystals could be detected using the 1050 cm^-1^ mode in CPDD rats (Figure 4B). Moreover, in the mixed rat model MSU and CPPD could be separately identified (Figure 4C), based on the SRS spectral differences between MSU, CPPD and bare tissue (Figure 4D-E). These results indicate that SRS is able to differentiate gout from pseudogout with high chemical specificity and may also be potentially applied in other crystallization diseases.

Note that previous tests on this gout model usually focused on changes in inflammatory factors and inflammatory cells, histopathology examinations of acute synovitis often failed without the presence of MSU deposition 32, 33. This is also shown in our imaging results comparing SRS and H&E on the same tissue. While SRS could successfully detect MSU microcrystals in fresh unprocessed tissue specimens (Figure 4), H&E sections from the same tissue specimen demonstrated negative results (Figure S7), which might due to the loss of MSU microcrystals (washed out or dissolved) during tissue processing with formalin fixation. SRS may hence provide more accurate approaches for early diagnosis of gout.

### Imaging MSU microcrystals in human synovial fluid and tophi specimens

**Figure 5:**
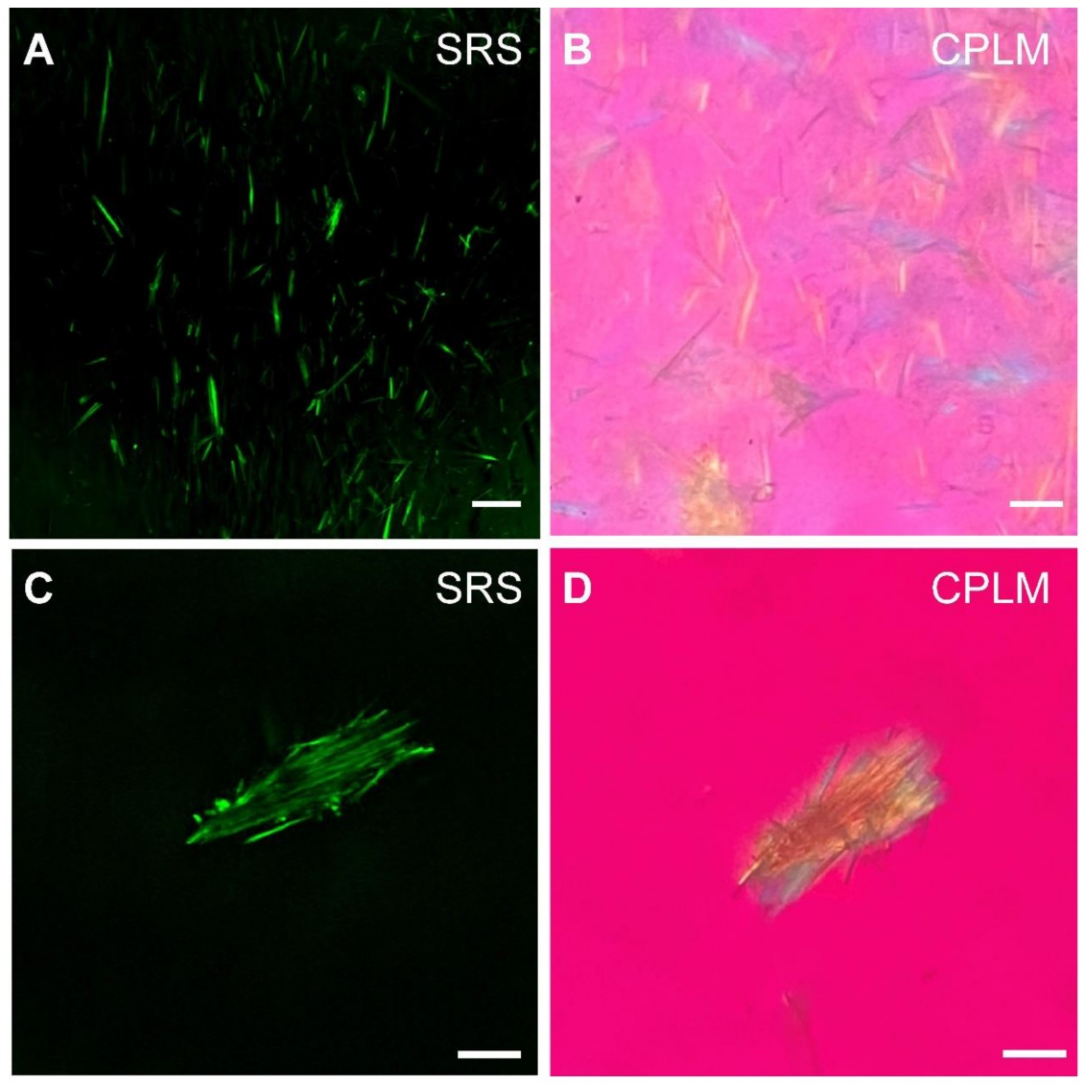
**Detection of MSU in synovial fluid of GA patients.** MSU microcrystals in the synovial fluid of patients with chronic gout arthritis imaged by **(A)** SRS and **(B)** CPLM. MSU crystal bows in synovial fluid imaged with** (C)** SRS and **(D)** CPLM. Scale bar:10 μm.

**Figure 6:**
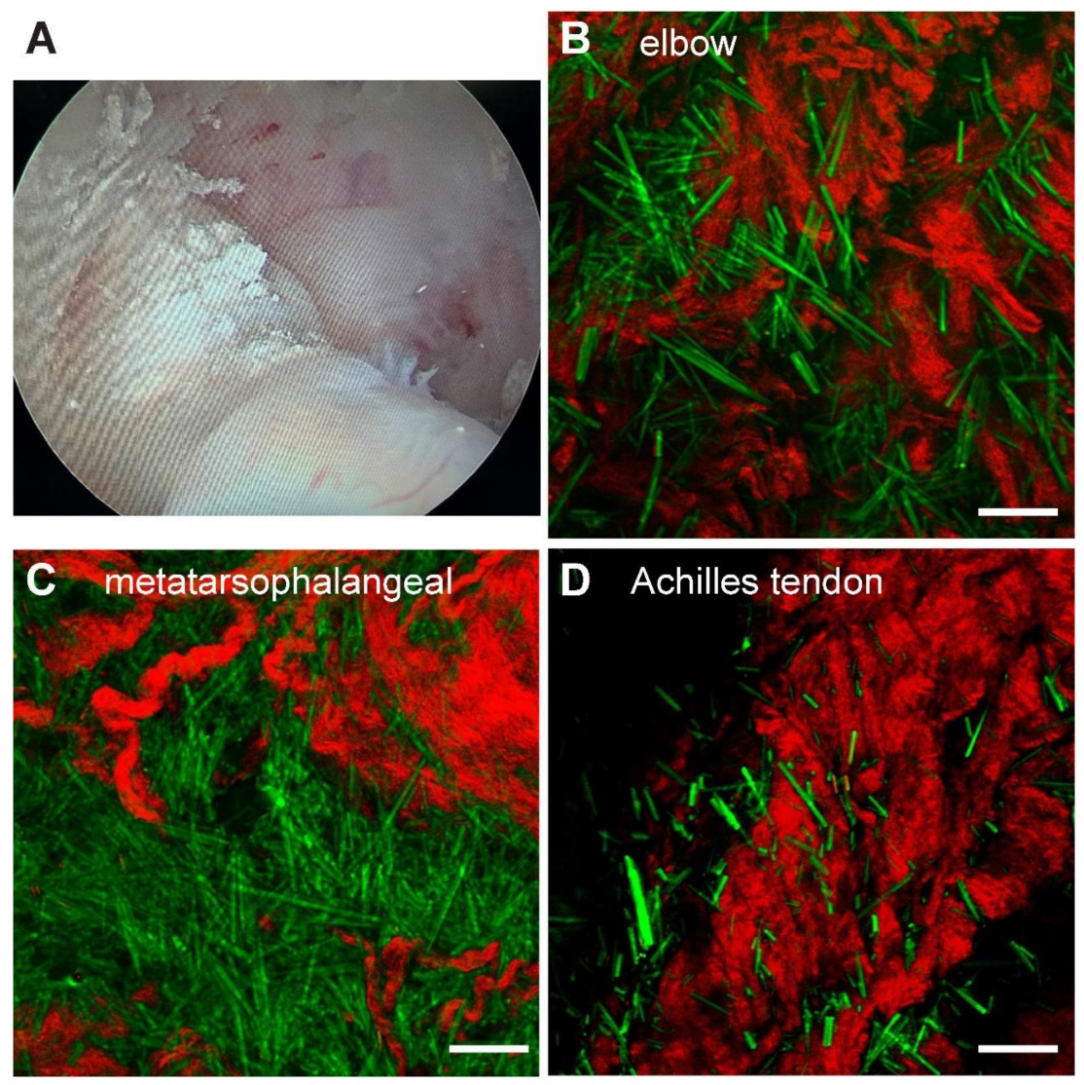
**Rapid diagnosis on fresh human surgical tissues of GA patients. (A)** Chalky tissues were harvested under arthroscopy. SRS images of unprocessed fresh tissues revealed intact MSU (green) depositions from **(B)** the elbow joint (n = 1), **(C)** the first metatarsophalangeal joint (n = 1) and (D) Achilles tendon (n = 1). Scale bar:10 μm.

We next evaluated the potential of SRS for rapid diagnosis and imaging of gouty arthritis using synovial fluids (Figure 5) and fresh human surgical specimens (Figure 6). MSU microcrystal in drops of synovial fluid aspirate of patients with chronic gout arthritis were imaged with SRS and CPLM. While CPLM revealed the crystal birefringence of MSU with changing colors reflecting different aligntment angles between light polarization and crystal axis (Figure 5B and D), SRS demonstrate more direct chemical specificity and quantitative meaures based on the Raman signature with the signal intensity proportional to molecular concentration (Figure 5A and C). These demonstrated that SRS is capable of detecting MSU in synovial fluid with single-crystal sensitivy and vibrational specificity.

Surgical tissues were dissected under arthroscopy from 3 patients diagnosed with hyperuricemia combined with joint lumps. The fresh specimens were briefly trimmed and placed directly between two coverslips and a perforated glass slide (0.5 mm thickness) for SRS imaging without further processing. We tested samples from multiple joint sources, including the elbow (*n=*1), first metatarsal joint (*n=*1) and ankle joint (*n=*2). All recruited patients were diagnosed as gout from the existence of large amount of MSU deposits in the tissues (Figure 6B-D). The raw images of SHG and SRS, as well as the method to extract the distributions of collagen fibers and MSU is shown in Figure S8. Each of these single images were taken in ~ 1 s, and large-area (centimetre-sized) specimen could be imaged within a few minutes with proper stitching and mosaicking techniques 34, 35, providing opportunities for rapid intraoperative diagnosis. Whereas the standard histopathology requires a time-consuming process of fixation, staining and then diagnosed by professional pathologists, which usually takes a few days and is difficult to adapt in the intraoperative settings.

**Figure 7:**
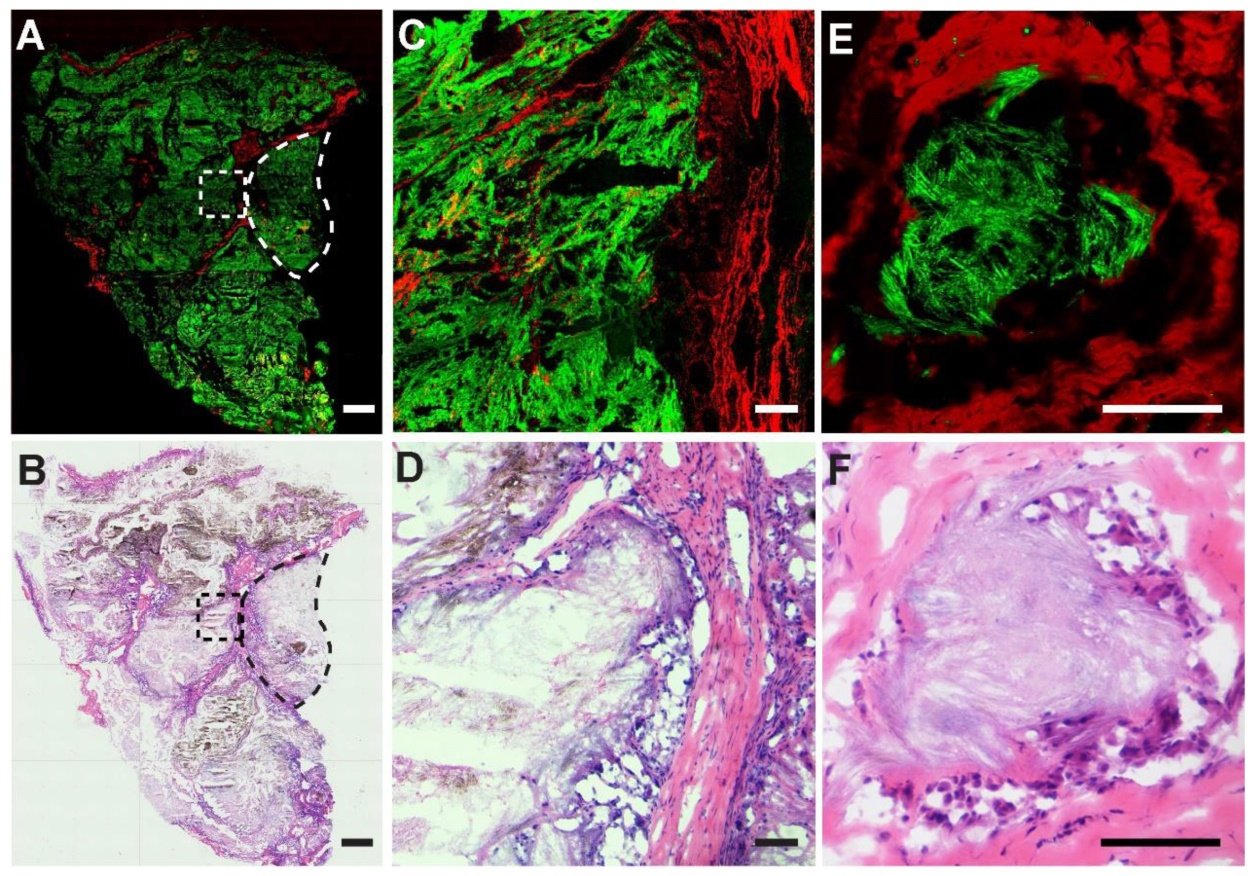
** Imaging thin frozen sections of human tissues. (A)** Representative stiched large-scale SRS/SHG image and **(B)** H&E image of adjacent tissue sections, showing the distributions of MSU (green) and collagen fibers (red), with a typical granuloma-like structure (dashed circle). **(C-D)** Enlarged images of the dashed square area in (A-B). **(E-F)** A typical tophi structure containing an enveloped crystal core. Scale bar: 500 μm (A-B), 100 μm (C-F).

We also performed SRS imaging on frozen tissue sections to compare with H&E on adjacent sister sections. A typical SRS/SHG image of a large area tissue section is shown in Figure 7A, demonstrating overall histoarchitectures that agree with traditional histology (Figure 7B), including collagen rich fiber-vascular areas (red) and MSU depositions (green). A typical granuloma-like sturcture enclosed by collagen fibers comprisingenveloped MSU crystals and dead immue cells could be readily visualized (dashed circles) and enlarged tissue morphologies are shown in Figure 7C-D. Detailed structures of a NETs structure in tophi are clearly demonstrated with an MSU core surrounded by collagen fibers (Figure 7E), consistent with the corresponding H&E result (Figure 7F) where MSU deposits were only slightly stained by eosin. It could be noticed that SRS is advantageous in detecting MSU based on the specific Raman identity, in contrast to the morphology-based judgement of MSU in H&E staining where MSU could not be specifically labeled. In addition, high-contrast SRS images provide a clearer pattern of the margin between granuloma-like masses and collagen fibers than traditional staining, which is necessary for precision cleavage and protection of healthy tissue in surgical treatment.

### Quantitative measurements of MSU in chronic gout

**Figure 8:**
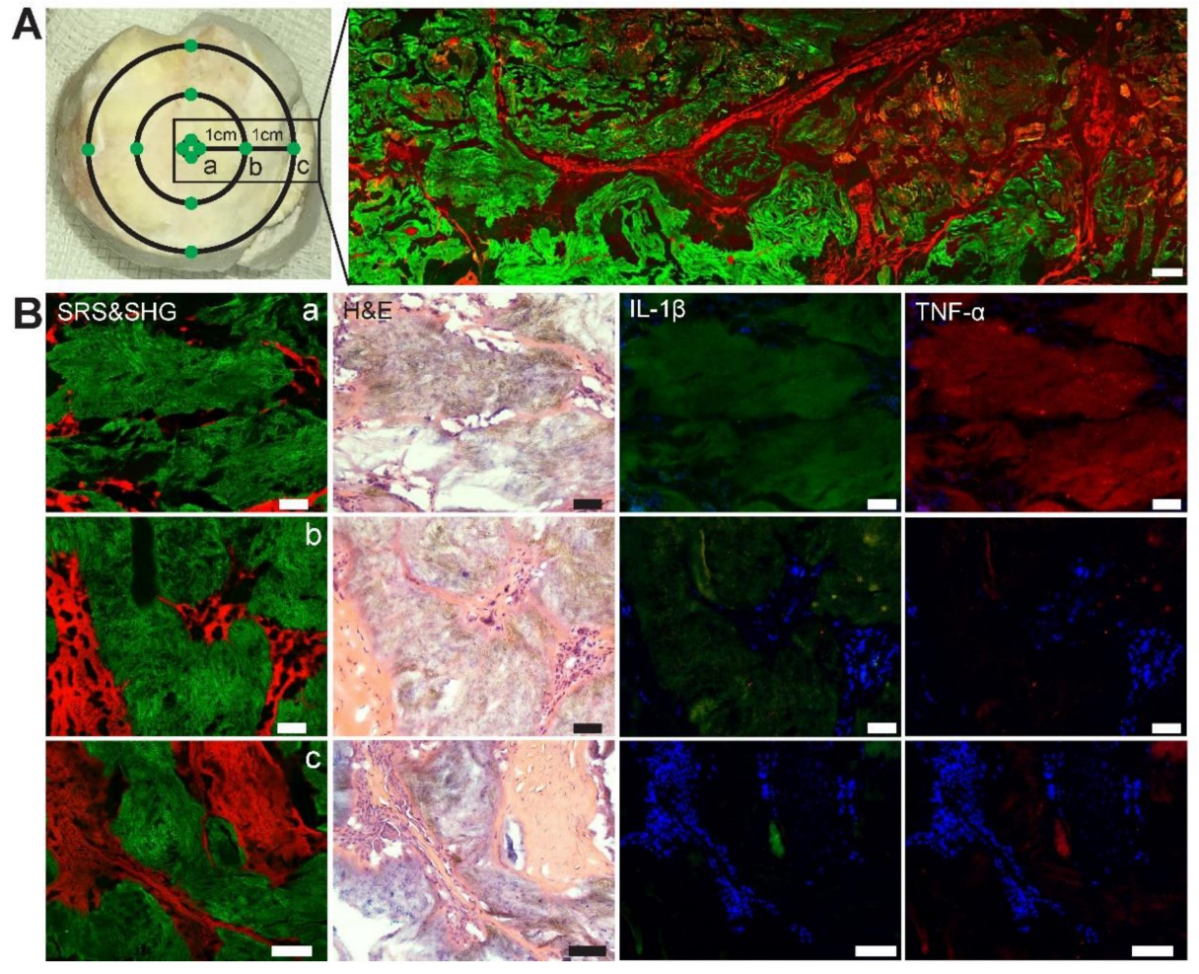
** Evaluation of chronic gout tissues with SRS, H&E and immunofluorescence. (A)** Tophus tissue of GA patients were sampled from three groups: center, 10 mm and 20 mm away from the center, with a large-scale SRS/SHG image (right) covering the rectangular area (left).** (B)** Representative images of SRS, H&E and immunofluorescence in the tissues (*n =* 120) to show the differences between the three groups. SRS/SHG images show MSU (green) and collagen fibers (red), while immunofluorescence images show cell nucleus (blue), IL-1β (green) and TNF-α (red). Scale bar: 500 μm (A), 50 μm (B).

We then tested whether SRS microscopy is capable of quantifying MSU deposition in human chronic gout tissues. Quantitative analysis of SRS has been commonly adapted based on the property that SRS intensity is linearly proportional to molecular concentration 14. Assuming that different distances from the center of tophi represent different pathophysiological stages of chronic GA, two gout specimens in regular ellipse with a radius of 10.5±0.5mm were selected for the study (Figure 8A). Specimens were dissected from three groups of regions classified by the distance from the center of the tophi: (a) central tophi; (b) 10 mm and (c) 20 mm from the center. For each specimen, three adjacent thin tissue sections were sliced and imaged with SRS, H&E and immunohistochemical staining, respectively. Representative images of the three modalities are shown in Figure 8B. While they demonstrated similar microstructures of gouty frozen sections that correlated well with each other, they also offered different information. SRS reflects the concentration and area of MSU deposition. H&E mainly reveals distributions of MSU, recruited inflammatory cells, and collagen. Whereas immunohistochemical results represent the distributions and expression levels of pro-inflammatory cytokines, including Interleukin-1β (IL-1β) and tumor necrosis factor-α (TNF-α), which have been known to be important signal molecules in the pathway of MSU-induced inflammation.

**Figure 9:**
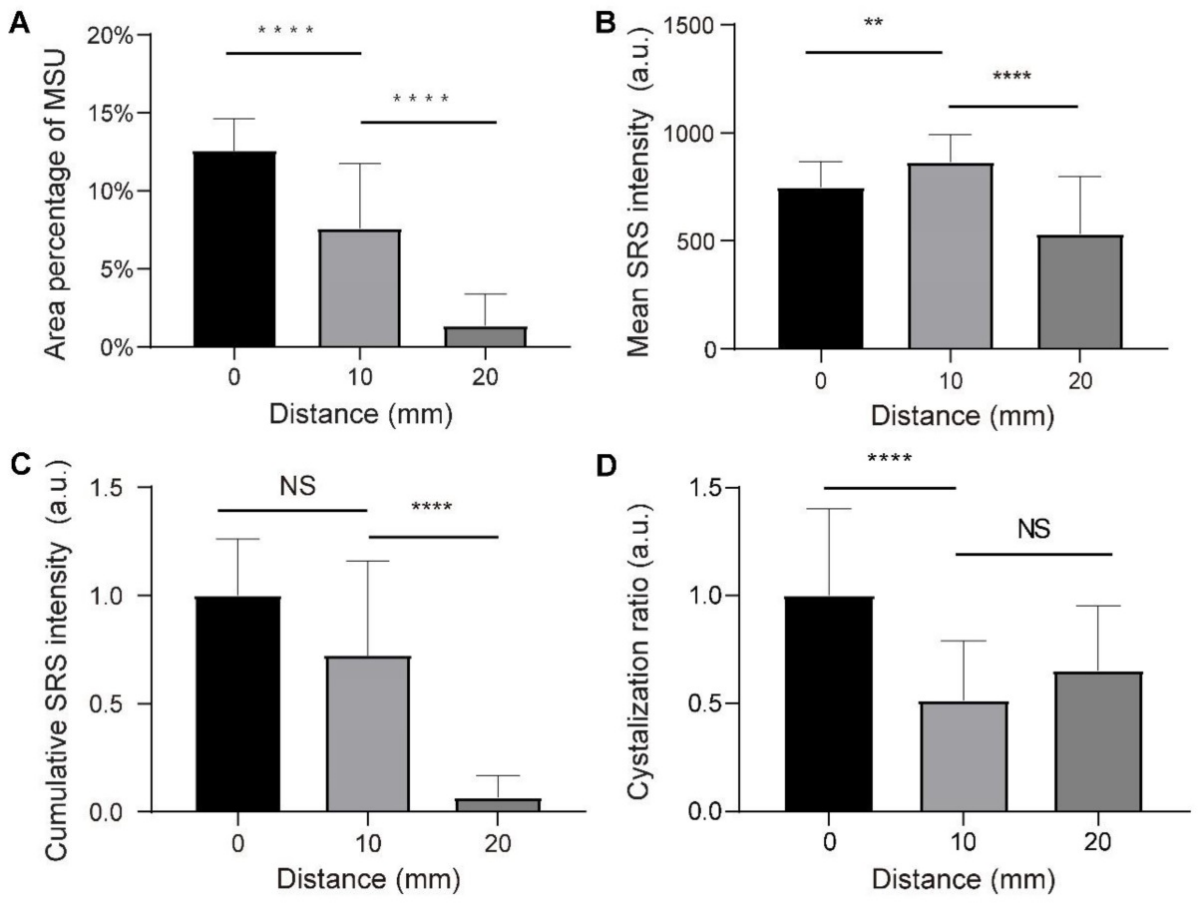
** Quantitative analysis of chronic tophi tissues from the three groups defined in Figure 8. (A)** Area percentage of MSU in each FOV. **(B)** Mean SRS intensity of MSU (per pixel) in each FOV. **(C)** Cumulative intensity of MSU in each FOV. **(D)** Crystallization ratio of MSU in each FOV. *N =* 40 for each group, Kruskal-Wallis test followed by Dunn's multiple comparisons test, ^****^P < 0.0001. Data shown as mean ± s.e.m.

We randomly selected 120 fields of views (FOVs) of 212 µm×212 µm from the three groups, and measured the dependence of various parameters on the distance to the tophi center. The area percentage of MSU was quantified by calculating the mean percentage of pixels in the MSU channel of SRS FOVs. It can be seen that the extent of MSU formation increases monotonically from the 20 mm distance towards the core (0 mm) (Figure 9A). On the other hand, the concentration of MSU (represented by the mean SRS intensity per pixel) shows a different pattern: it first increases from the 20 mm towards the closer peripheral (10 mm), but slightly drops at the core (Figure 9B). The content of MSU was quantified as the normalized cumulative SRS intensity of MSU, which shows a similar pattern as the area of MSU (Figure 9C). In addition, crystallization ratio of MSU was measured as the ratio between the number of pixels in SHG and SRS channels within MSU areas, which appeared significantly higher in the core region (Figure 9D). Moreover, the expression levels of IL-1β and TNF-α were represented by the mean intensity of the immunofluorescence images, showing similar patterns as the area of MSU (Figure S9A-B).

These observations indicate that SRS may provide a convenient means to quantitatively measure MSU in terms of area, concentration, content and crystallization ratio. Their spatial distributions were found to be consistent with the general recognition of the pathophysiological process of tophi. The depositions of MSU tend to recruit a large number of neutrophils in acute phase, which then ingest MSU crystals, activate inflammasomes and produce large number of pro-inflammatory factors through the IL-1β-dominated signaling pathway 28. At histological level, typical characteristics are described as a particular form of cell death known as NETosis and then the formation of neutrophil extracellular traps (NETs) and, which densely pack MSU and degrade cytokines, especially in the center zone of the tophi. Meanwhile, the outer structure of tophi is surrounded by newly formed fibrovascular zone where a combination of acquired immune cells and anti-inflammatory factors interact and lead to remodeling 36. These agree well with the distribution of MSU area, content and crystallization ratio, as well as the expression levels of IL-1β and TNF-α, demonstrating the common increase-towards-the-core pattern. On the other hand, the formation of NETs densely envelops MSU microcrystals, which explains the counter-intuitive distribution of MSU concentration, with a decrease in the fully developed tophi core compared to that of the peripheral region.

**Figure 10:**
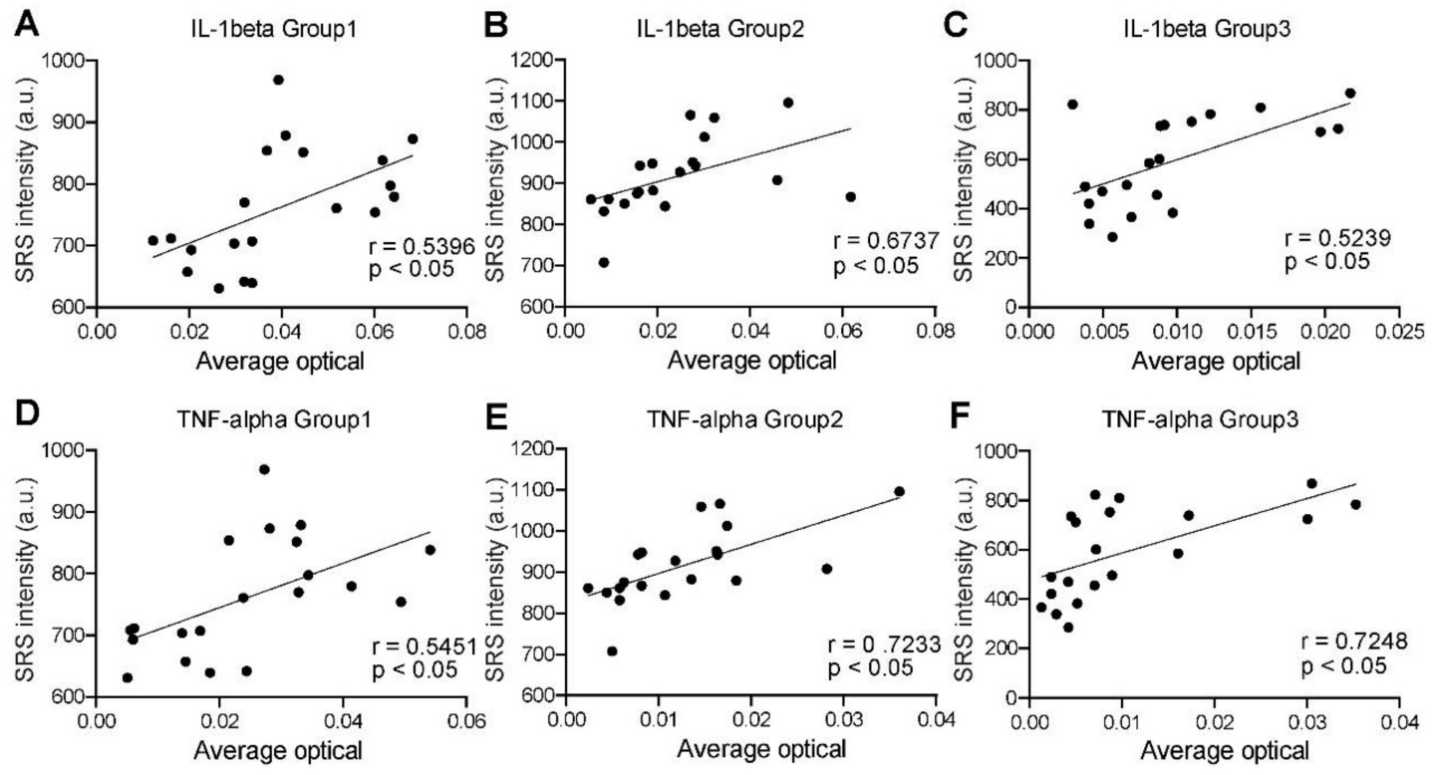
** Correlation between SRS microscopy and immunofluorescence in corona zone. (A-C)** Correlation between SRS intensity of MSU and IL-1β (*n=*40 for each group; Pearson r test was applied in group 1; Spearman r test was applied in group 2 and 3, R = 0.5396, 0.6737, 0.5239, respectively; P <0.05, calculated by two-sided t-test.).** (D-F)** Correlation between SRS intensity of MSU and TNF-α (*n=*40 for each group; Pearson r test was applied in group 1; Spearman r test was applied in group 2 and 3, R = 0.5451, 0.7233, 0.7284, respectively; P <0.05, calculated by two-sided t-test.).

Finally, we analyzed the correlations between SRS and immunofluorescence intensities to quantify the distribution pattern of IL-1β and TNF-α expression levels in the three groups. As mentioned above, proinflammatory factors are expressed differently in the non-cellular and cellular regions. Cytokines are degraded by NETs in the MSU deposition region, while the collagen region is dominated by fibroblasts and plasma cells. Previous studies have suggested that the pathophysiological process in the coronal zone is similar to that of an acute flare phase 28. Therefore, the expression of these two representative cytokines is most significant and homogeneous in the coronal region (defined as the transition zone between the dense tophi core and the surrounding fibrous vascular region) (Figure S9E). Our preliminary statistics also found that the expression in this region was higher and more consistent with the normal distribution (Figure S9C-D). A total number of 60 pairs of adjacent SRS and immunofluorescence images were randomly selected in the corona zones. Statistical results were then generated by correlating their intensities at the pixel level. For all the groups, both IL-1β and TNF-α expression levels showed moderate positive correlations with SRS intensity of MSU, with correlation coefficients (R values) above 0.5 and P value less than 0.05 (Figure 10). Such result is consistent with the NETs theory, implying that SRS intensity of MSU may predict the degree of inflammation in the corresponding area. Therefore, SRS is capable of quantitatively characterizing various properties of tissue specimens, and analyzing tophi formation following cascade reactions.

## Discussion

We have demonstrated the advantages of SRS microscopy for histological identification of gout without tissue fixation or staining. The results were verified in various types of tissues, including synovial fluid, synovium, tendon and tophi from both rat models and different joints of human. Sensitive and chemical specific detection of MSU crystals were realized, even in cases where traditional histopathology might have failed, as shown in the early diagnosis results of rat acute gouty arthritis model. In previous studies on rat gouty arthritis model, histopathological examinations tend to show the absence of MSU depositions due to limited dose of MSU and metablism by uricase 37. Our study demonstrated that SRS has the ideal diagnostic capability and sensitivity in fresh tissues, even at the early stage of gouty arthritis.

It is also worth comparing SRS with another popular Raman enhancing technique known as surface-enhanced Raman scattering (SERS). SERS has the unique capability of detecting low concentration molecules with extremely high sensitivity, and has been investigated in gout diagnosis, with SERS nanoparticles probing uric acid in human body fluids include synovial fluid or tear fluid 38. However, the imaging capability (sensitivity and speed) of SERS is largely limited so far. In addition, compared with SRS, application of SERS was also limited by biocompatibility of metal nanoparticles, slow point measurements, sample preparation, and it could not provide quantitative information of insoluble species in tissues 39, 40. On the contrary, the formation of MSU depositions ensures high local concentration that allows SRS to generate sufficiently strong signals to image at high speed. Moreover, SRS microscopy has the advantage of background-free detection, whereas spontaneous Raman suffers from autofluorescence, and CARS suffers from non-resonant background 14, 41. Additional advantages of SRS include the enhanced tissue penetration depth using near-infrared lasers 42, as well as the intrinsic optical sectioning capability for three-dimensional imaging 30.

The finding that SRS could provide intraoperative and differential diagnosis of gout and pseudogout has important clinical value. Intraoperatively, arthroscopy observed that the MSU is not only enveloped at the first metatarsophalangeal joint, elbow joint and the finger joint, but also deposited at the atypical areas (such as the Os trigonum or the Achilles tendon etc.) with different shapes and sizes. Our study confirmed that SRS is able to diagnose gout in a variety of different joints and demonstrate the distribution relationship between MSU deposition and surrounding tissues A good consistency has been reached between traditional histopathology and MSU channel of SRS microscopy. For refractory gout, surgery often remains a last resort of debulking tophi, yet it still suffers from complications caused by inaccuracy (such as damage to normal structure) due to the lack of real-time evaluation methods 43. Although the imaging depth of SRS is limited by optical aberration of tissue (~ 200 µm), it has shown potentials in rapid intraoperative diagnosis both *in vivo* and *ex vivo*, by point-checking the resection cavities 25, or quick evaluation of fresh surgical tissues 16, 26. With the development of SRS detection techniques such as coherent Raman endoscope 44, it may provide new approaches to trace MSU *in vivo* when combined with arthroscopy to provide adequate quantitative information of MSU depositions intraoperatively, which could offer guidance to optimize surgical strategies and assist decision making, especially on resection margins with maximum removal of MSU deposition and effective preservation of normal structures. Moreover, SRS microscopy integrated with deep-learning algorithms could potentially realize rapid and accurate intraoperative gouty diagnosis, which is among our further work plans 26, 45.

Our study quantified the characteristics of MSU deposition in tophi and correlated it with the level of inflammation. Researches on the interactions between various physicochemical and biological factors (PH, temperature, concentration of ions or proteins) on the solubility, nucleation and growth of MSU have attracted increasing attentions 46. It has been found that a series of biological factors (such as collagen, proteoglycan, cartilage factors, antibodies, etc.) can regulate the crystallization of MSU and ultimately lead to the differentiation of the disease process 47, 48. SRS may provide quantitative evaluation and specific imaging of MSU to reveal the relationship between these factors and crystal growth. Further studying the* in vivo* process of MSU crystallization and the interactions with immune system in animal models may also become possible.

In summary, we have demonstrated that SRS microscopy could provide label-free and chemical specific detection of MSU, enabling early detection and rapid diagnosis of gout in various types of tissues and joints. Exploiting the vibrational fingerprints of MSU and CPPD, SRS is able to precisely differentiate gout from pseudogout. Furthermore, quantitative SRS measurements of MSU in gout tissues were found to correlate well with the pathophysiological process of tophi. Our study may open up new opportunities for intraoperative diagnosis of gout, as well as fundamental biomedical researches on the formation and development of MSU in various MSU-based diseases.

## Materials and Methods

### Human specimen collection

For Synovial fluid analysis, we performed identification of MSU crystals in microscope slides with a drop of synovial fluid aspirate by SRS and CPLM (Olympus Corporation, Japan) with red plate compensator (*n=*2). We then collected intraoperative specimens of tophi or synovial tissue from different patients (*n=*4), All processes were approved by the Ethics Committee of Huashan Hospital with informed written consent (KY2020-060). The patients (all middle-aged men) were diagnosed with hyperuricemia for more than five years and had limited mobility due to a joint mass and met surgical indication after evaluation. Intraoperative specimens were collected from the elbow (*n=*1), first metatarsophalangeal (*n=*1) and ankle (*n=*2). In the part of quantitative diagnosis, two intact enveloped tophi were divided into three groups according to the distance from the center of tophi (0 mm, 10 mm and 20 mm). For each group, four specimens were dissected with equal size (3 mm×3 mm×3 mm). The specimens (*n=*12) were fixed in 4% paraformaldehyde solution for 24 hours and embedded in OCT-Freeze medium. For each specimen, 10 groups of sections were made, each consisting of three consecutive 8-μm-thick sections. For each group of sections, three consecutive sections were then imaged by SRS microscopy, and optical microscope after H&E staining or immunofluorescence double-label staining (Figure 1).

### Animal Study

A total of nine male SD rats aged 8 weeks (Shanghai Lab. Animal Research Center, Shanghai, China) were used for the present study. Experimental procedures passed a review by the Animal Welfare and Ethics Group, Department of Experimental Animal Science; Shanghai Medical College of Fudan University, Shanghai, China (Approval Number: 2019020405). The MSU crystal (Sigma Chemical, Co.St.Louis, MO, USA) was prepared by PH titration of uric acid according to the method proposed by previous study 49. The injection of MSU was performed as previously described 49. On day 0, nine rats were randomly separated into three groups. Inhalation of 3% isoflurane was used to induce anesthesia, and inhalation of 1.5% isoflurane was used to maintain anesthesia. A 3 mm incision was made by scalpel on the lateral side of the knee joints. MSU suspension, CPPD (Sigma Chemical Co, St.Louis, MO, USA) suspension and fifty-fifty mixed crystals suspension of 50 mg ml^-1^ was injected into joint cavity at 3 ml in three groups, respectively. On day 3, the rats were sacrificed, and the synovium of joint cavity was dissected and harvested for further analysis.

### SRS microscopy system

In spontaneous Raman scattering, one laser beam at a frequency ω_p_ illuminates the sample and the signal is generated at the Stokes and anti-Stokes frequency, respectively, due to inelastic scattering. In SRS, however, due to stimulated emission, two laser beams at ω_p_ and ω_s_ coincide on the sample. When the difference frequency, Δω= ω_p_-ω_s_ matches a particular molecular vibrational frequency Ω, amplification of the Raman signal is achieved and SRS boosts vibrational excitation by a factor of 10^7^ 14. The setup of our SRS imaging system is illustrated in Figure S3. We used commercial pulsed femtosecond (fs) laser beams from optical parametric oscillator (OPO, Insight DS+, Newport, CA) with dual outputs as the light source. The fundamental 1040 nm laser was used as Stokes beam (~150 fs), and the tunable OPO output (690-1300 nm, 120 fs) was used as pump beam. The femtosecond pulses were chirped through high dispersive glass rods (SF57) to several picoseconds (pump: ~3.8 ps, Stokes: ~1.8 ps) to realize spectral focusing obtaining high-resolution spectral which is identical to spontaneous Raman spectral, in which we can set time-delay between pump beams and Stokes to get accurate Raman shift 50. And we scan time-delay between two beams to get SRS spectral. We set the wavelength of pump laser as 976 nm to match the peak (630 cm^-1^) of MSU in spontaneous Raman spectral (Figure 2B). For SRS imaging of CPPD, we set the wavelength of pump laser as 937 nm to match the Raman peak (1050 cm^-1^) of CPPD. Typically, 801-nm pump laser was used to imaging lipid/protein. The Stokes beam was intensity modulated by an electro-optical modulator (EOM) at 10 MHz and aligned with pump beam through a dichroic mirror (DMSP1000, Thorlabs). The SRS signal intensity of MSU in images is rely on polarization of laser beams (Figure S5), in which the polarization dependence diagram was acquired by scanning a half-wave plate with a step of 2 degree. In order to depress the effect of polarization of laser beams, we used a 1/4-wave plate to make linear polarization beams be circular polarization beams. The aligned beam was delivered to the laser scanning microscope (FV1200, Olympus) and focused onto the gout samples through an objective (UPLSAPO 60XWIR, NA 1.2 water, Olympus). The stimulated Raman loss (SRL) signal generated by MSU in gouty samples was filtered with a band-pass filter (CARS ET890/220, Chroma) to eliminate the Stokes beam and detected by a home-built back-biased photodiode and demodulated with a lock-in amplifier (HF2LI, Zurich Instruments) to get SRS signal in each pixel. By scanning the laser using two galvo mirrors, an SRS image is generated. Simultaneously, we performed epi mode to detect SHG signal excited by the Stokes beam (1040 nm) with a photomultiplier (PMT) through a narrow band-pass filter (FF01-520/10, Semrock), which generate SHG images to show the distribution of collagen fiber in gouty tissues. The SRS signal of MSU is relative weak compared with lipid or protein, the optical power of the pump and Stokes beams at the sample were kept around 80 mW and 160 mW. The size of each field of view is 512 × 512 pixels (180 × 180 µm) with lateral resolution of ~ 350 nm, and the pixel dwell time is 2 µs.

### Spontaneous Raman spectroscopy

The spontaneous Raman spectra was measured by a home-built Raman spectrometer, including a monochromator (iHR320, Horiba), a charge-coupled device camera (Symphony, Horiba), and a microscope (IX71, Olympus) with a 40X objective. A 633 nm helium-neon laser beam excited spontaneous Raman scattering of MSU and CPPD standard samples. In order to get Raman spectral of lipid and protein, we measured the Raman spectral of OA (Oleic acid) and BSA (bovine serum oleate) which can represent the distribution of lipid and protein in human body. Moreover, quartz cover slips were used to cover MSU crystal which can depress autofluorescence generated by normal glass.

### Imaging process and quantitative analysis

All steps of image process used ImageJ. For gouty tissues, firstly, we let SHG images subtract SRS images to extract the collagen fiber channel because both MSU crystal and collagen fibers contribute to SHG signal. Secondly, we can merge the MSU (green) and collagen fiber (red) channel processed previously into a two-color image so that the distribution of MSU and collagen fibers was shown clearly. The image process of CPDD model is similar to gouty tissues. For large-scale tissues imaging, Olympus software has an automatic mosaic imaging program and we form a full-sized image by merge each FOVs with custom written MATLAB program. Quantitative analysis of SRS and SHG images were performed with ImageJ. Firstly, we performed “adjust” and “threshold” function to set the threshold value of intensity profile to get proper MSU SRS signal and depress background. Average SRS intensity of MSU was calculated to analysis the density of MSU in each FOV because of the linear dependence of SRS intensity on chemical concentration 14. Then, we used “measure” to get the average intensity and area of MSU. Moreover, we analysis average SHG intensity of MSU to get a crystallization ratio because amorphous urate does not have SHG signal due to its non-crystal, which SHG signal intensity can reflect crystallization ratio sometimes. In order to conduct a correlation analysis between the corresponding images in successive 8-μm-thick sections of SRS and immunofluorescence, we adopted the coordinate system of the microscope stage for accurate spatial sampling and correspondence. All processes are performed and recorded by a blinded researcher.

### Histology

The synovium from patients and rats were processed for H&E staining and immunofluorescence staining as previous described 38, 51. The required reagents were purchased from Sigma Chemical Co.St.Louis, MO, USA and Servicebio, Wuhan, China. For immunofluorescent staining, the sections were subsequently incubated with the primary antibody. The antibodies to IL-1β (16806-1-AP:1:200) and TNF-α (60291-1-lg:1:200) were obtained from ProteinTech (Chicago, IL, USA). After incubation with CY3/FITC-conjugated secondary antibodies, the signals were visualized using fluorescent microscope (Eclipse C1, Nikon, Japan) and image system (DS-U3, Nikon, Japan) were used for imaging of samples.

### Immunofluorescence analysis

Using an immunofluorescence average optical density value (AO) analysis assay, TNF-α/IL-1β expression was quantified. At least 5 200 - fold fields were randomly selected in each group to be photographed. It was insured that the organization filled the entire field of view and the background light is the same for each photo. Image-pro Plus 6.0 software (Media Cybernetics, Inc., Rockville, MD, USA) was used to convert green/red fluorescence monochrome photos into black and white images, and then the unified standard for judging all positive photos was determined. The integral optical density (IOD) and pixel AREA of tissues of positive images were obtained by analyzing each photo. Average optical density value was calculated, and AO=IOD/AREA. The higher the AO value indicated the higher the positive expression level.

### Statistical analysis

Statistical analyses were performed by using SPSS 13.0 and GraphPad Prism 8.0.1 software. All datasets were tested for normality for t-test, and if the normality test failed, the Mann Whitney rank-sum test was used for intra-group comparison. For the comparison of SRS signal intensity, area and fluorescence AO, data were assessed for normality for one-way ANOVA, and Kruskal-Wallis test followed by Dunn's multiple comparisons test was used if the normality test failed. For correlation analysis, data were assessed for normality for Pearson r test, and Spearman r test was used if the normality test failed. Results are expressed as mean ± s.e.m. or median quartile. P < 0.05 is considered as significant.

## Supplementary Material

Supplementary figures.
