# Supplementary material for: Highly specific and label-free histological identification of microcrystals in fresh human gout tissues with stimulated Raman scattering
Source: Theranostics. 2021 Jan 1;11(7):3074–88. doi: 10.7150/thno.53755 (PMC7847673; doi:10.7150/thno.53755)
Supplement: Supplementary file 1 — Supplementary figures. [file thnov11p3074s1.pdf]

Highly specific and label-free histological identification of microcrystals in fresh human gout tissues with stimulated Raman scattering

Bohan Zhang[2#], Hanlin Xu[1#], Jun Chen[1], Xiaoxia Zhu[3], Yu Xue[3], Yifan Yang[2], Jianpeng Ao[2], Yinghui Hua[1]*, Minbiao Ji[2]*

[1]Department of Sports Medicine, Huashan Hospital, Fudan University, Shanghai 200040, China.

[2]State Key Laboratory of Surface Physics and Department of Physics, Human Phenome Institute, Multiscale Research Institute of Complex Systems, Academy for Engineering and Technology, Key Laboratory of Micro and Nano Photonic Structures (Ministry of Education), Fudan University, Shanghai 200433, China.

[3]Department of Rheumatology, Huashan Hospital, Fudan University, Shanghai 200040, China.

[#] These authors contributed equally.

***CORRESPONDENCE:** hua_cosm@aliyun.com, minbiaoj@fudan.edu.cn

Supplementary Materials

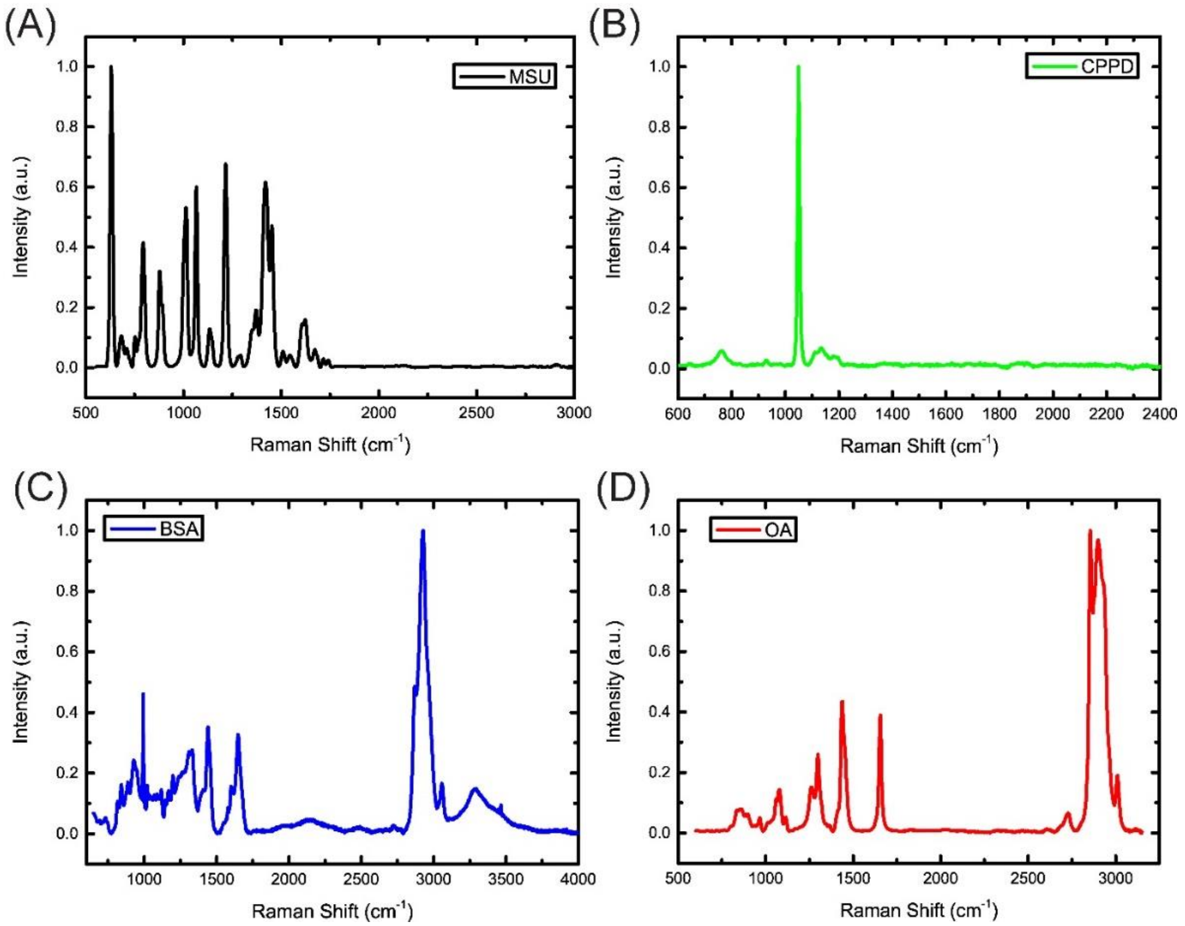

Fig. S1. **Spontaneous Raman spectra of standard chemicals. (A)** MSU, **(B)** CPPD, **(C)** BSA, **(D)** OA.

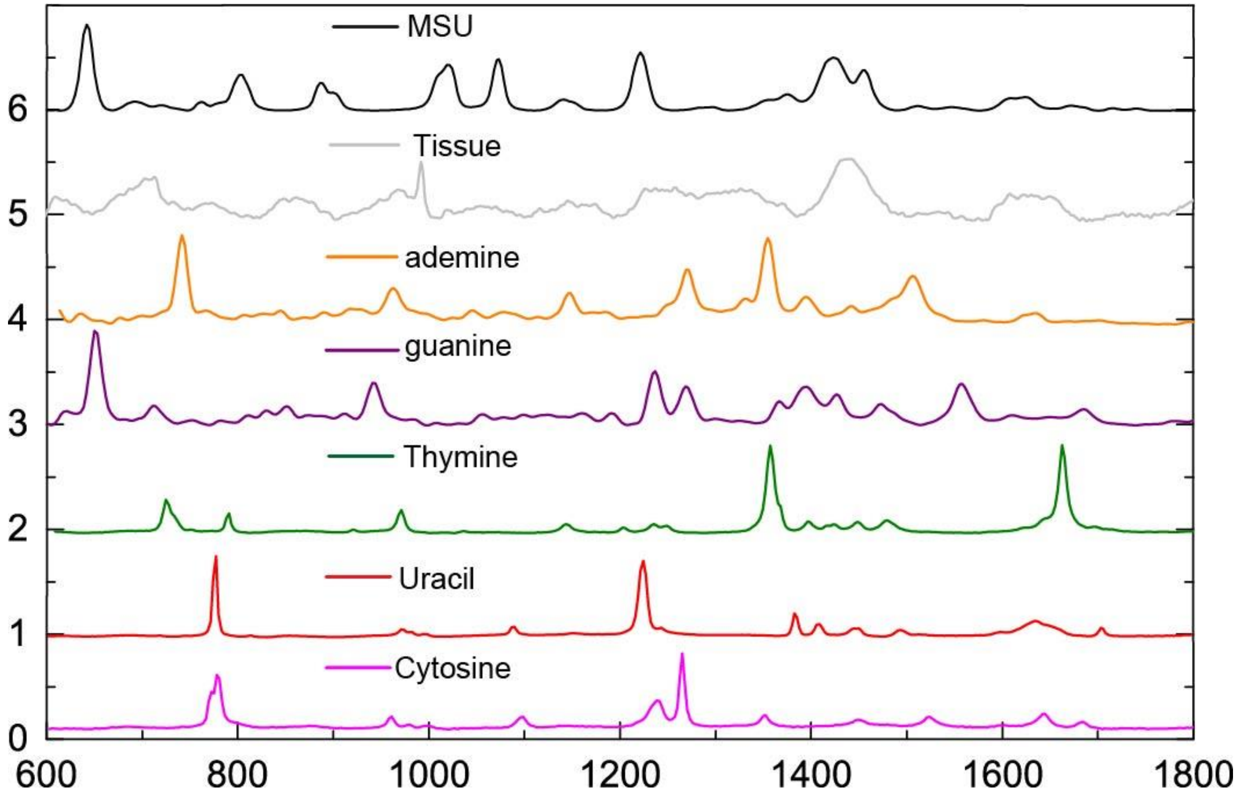

Fig. S2. **Spontaneous Raman spectra of MSU, bare tissue, purine and pyrimidine derivatives.** Note that although guanine has a Raman peak at ~ 650 cm^{-1}, it has much less local concentration than the MSU crystals in real tissue samples, and thus does not interfere with measurements for MSU.

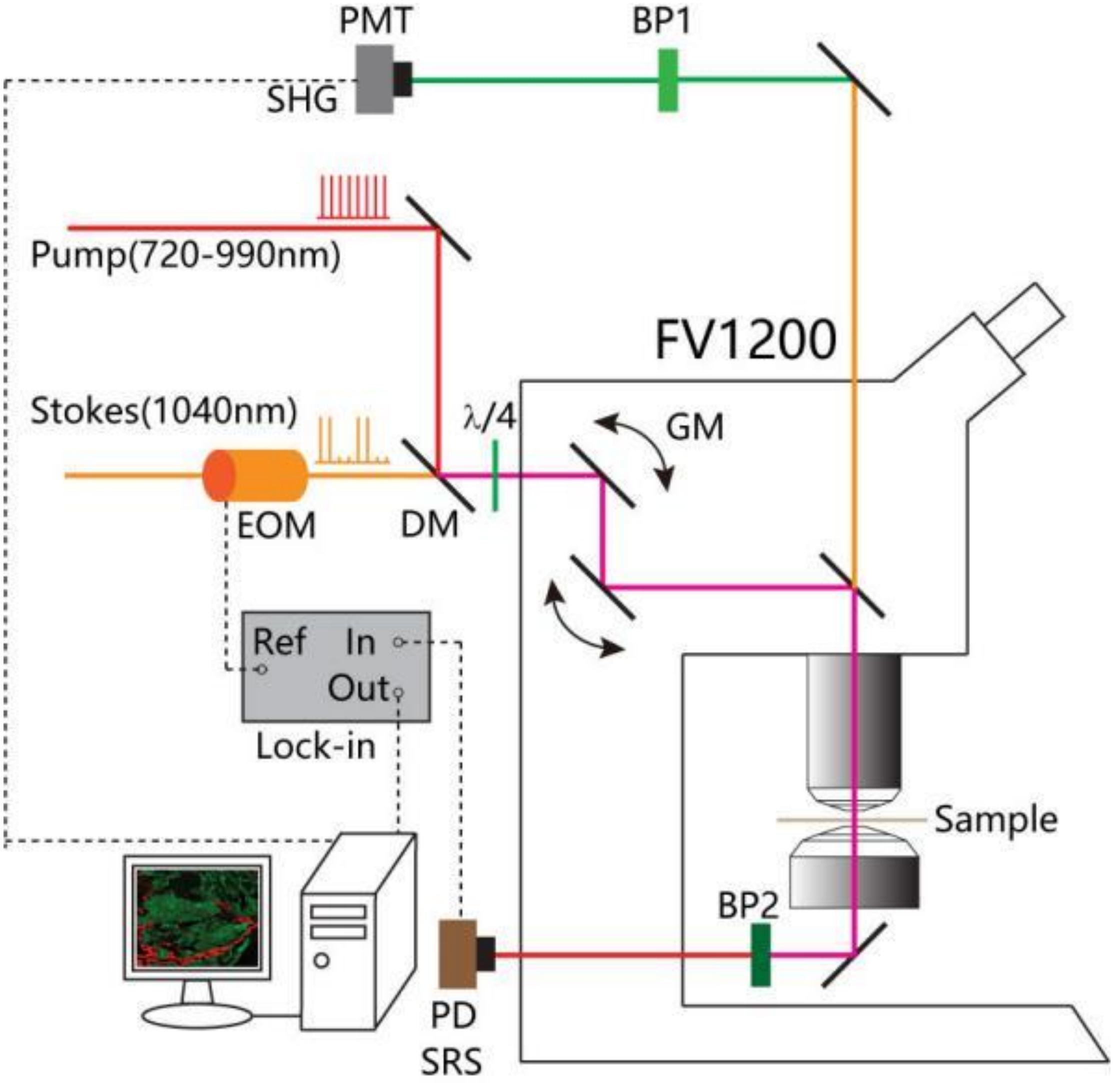

Fig. S3. **Optical layout of the SRS/SHG microscope.** EOM: electro-optical modulator; PD: photodiode; BP1: 520/10 band-pass filter; GM: galvo mirror; BP2: 890/220 band-pass filter; PMT: photomultiplier tube; DM: dichroic mirror; λ/4: quarter-wave plate.

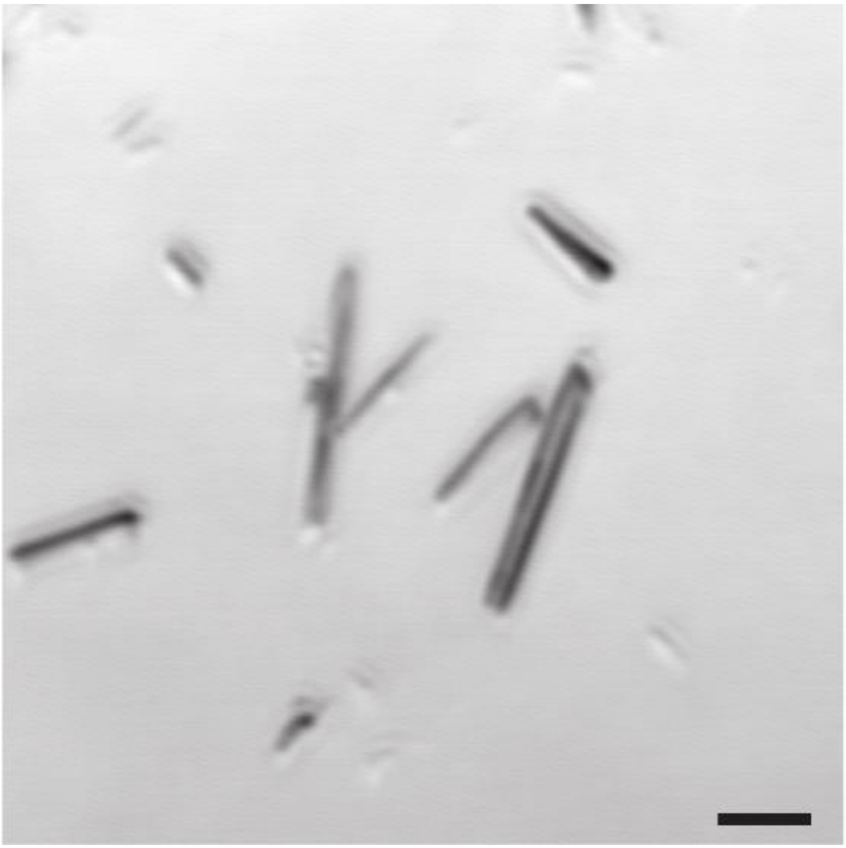

Fig. S4. Bright field image of typical MSU crystals shown in Fig. 3A-C. Scale bar: 5 μm.

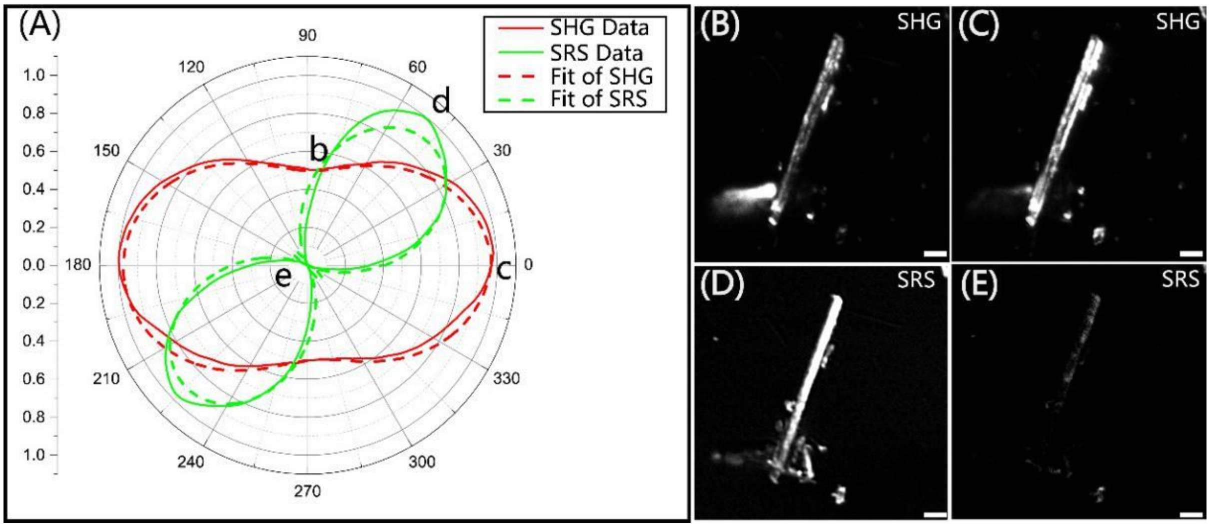

Fig. S5. Polarization-dependent SRS and SHG imaging in MSU crystals. (A) Polarization dependence curve of SHG and SRS signal (630 cm^{-1}) generated by MSU. **(B-E)** SHG and SRS images of polarization configurations at b, c, d, e, respectively. Scale bar: 3 μm.

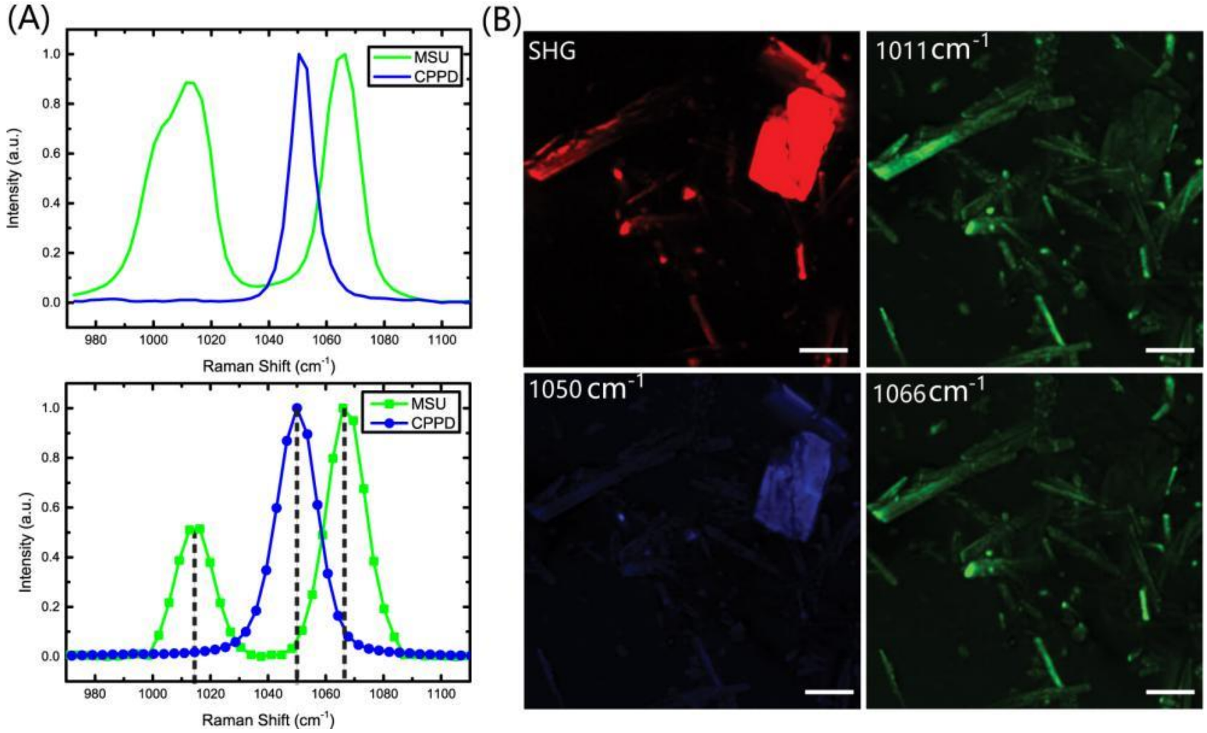

Fig. S6. Differentiation of standard MSU/CPPD samples with multicolor SRS. (A) Spontaneous Raman and SRS spectra of CPPD/MSU standard samples; **(B)** SRS/SHG images of CPPD/MSU mixed sample at selected Raman peaks. Red: SHG signal; green: SRS signal generated by MSU; blue: SRS signal generated by CPPD. Scale bar: 10 μm.

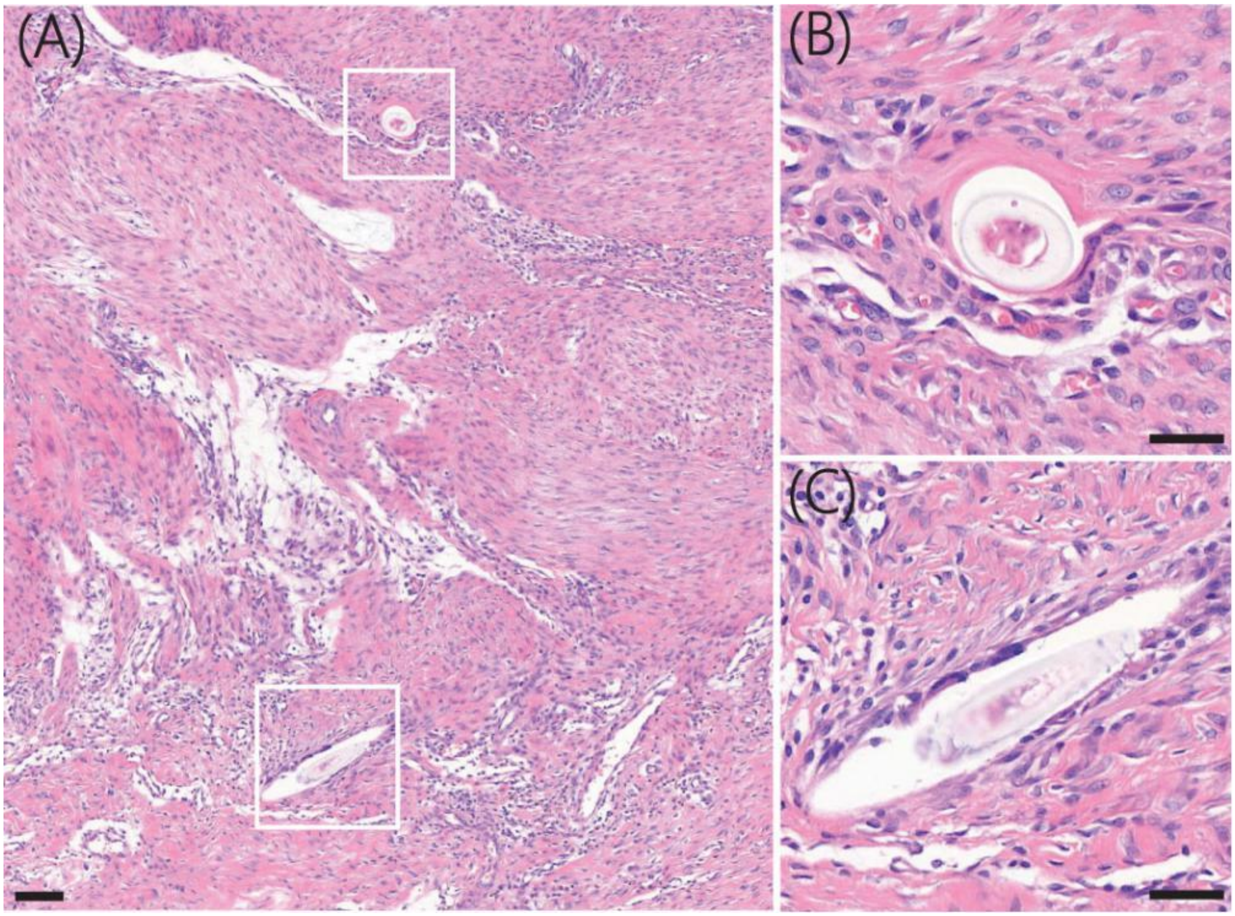

Fig. S7. Representative H&E images demonstrating synovitis on adjacent tissue sections. (A) Typical changes in synovitis were detected, including comprehensive synovial cell proliferation and acute inflammatory cell infiltration. **(B-C)** showed possible MSU deposition, but such changes were hard to detect and are not specific enough to make an accurate pathological diagnosis Scale bar: 100 μm.

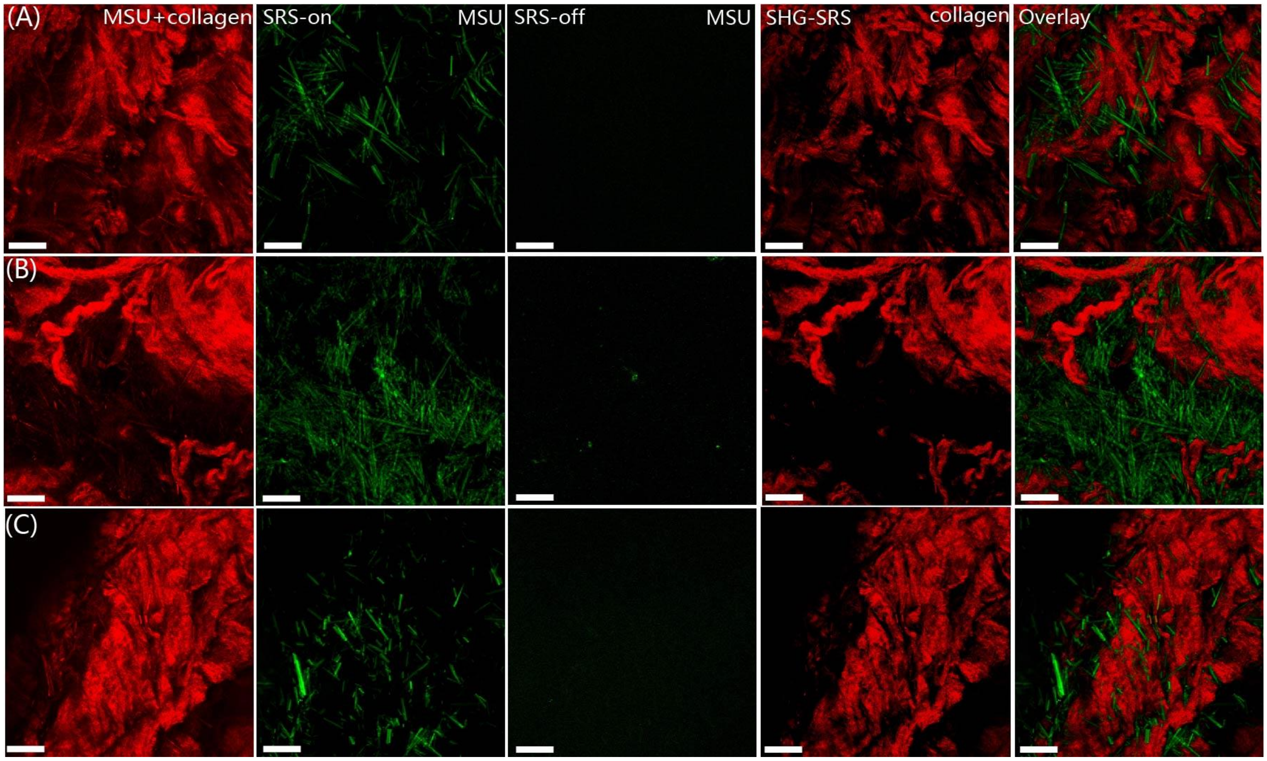

Fig. S8. Data processing of multimodal images of Fig. 5. (A) the elbow joint, **(B)** the first metatarsophalangeal joint, **(C)** Achilles tendon. The first column represents raw images of SHG, in which both MSU and collagen generated SHG signal; the second column represents raw data of SRS images of MSU; The third column represents off-resonance images of the same tissue. Collagen images were generated by subtracting SRS signal from SHG (fourth column). The fifth column are the overlay of MSU and collagen. Scale bar:10 μm.

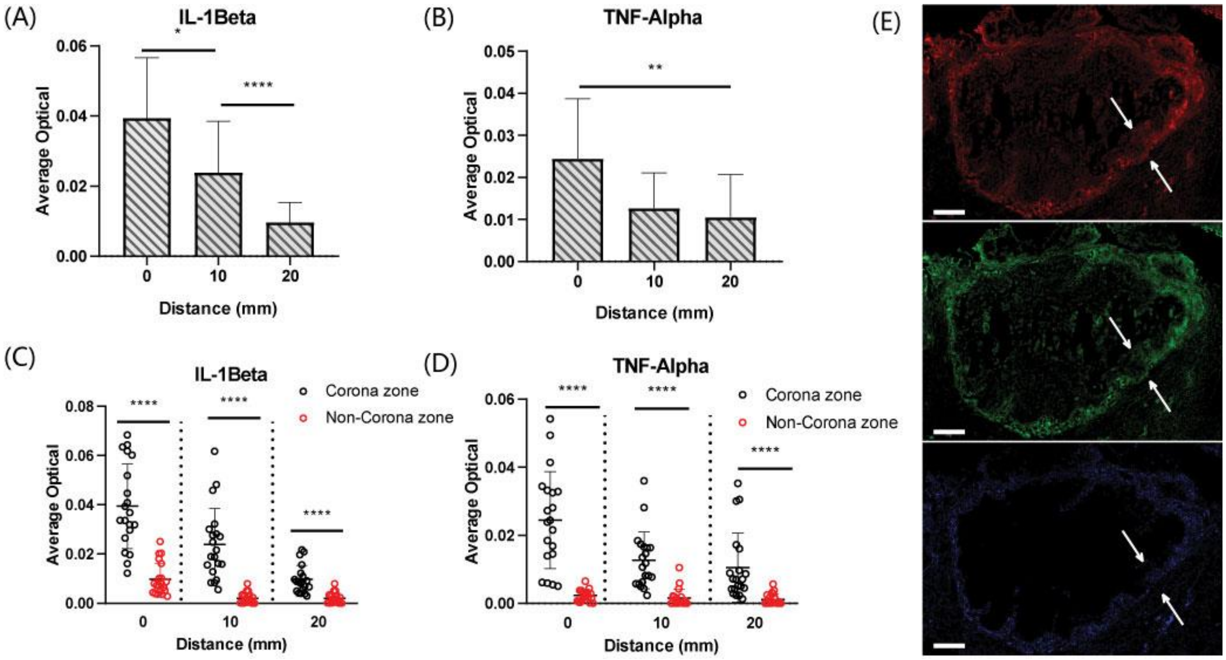

Fig. S9. Immunohistochemical inflammatory cytokines level in random field of view. Immunofluorescence sections revealed the average optical density of IL-1β **(A)** and the average optical density of TNF-α **(B)** in different groups. The area of average optical density of IL-1β decreases as the distance from the tophi center increases (*n*=20 for each group; Kruskal-Wallis test followed by Dunn's multiple comparisons test, *P=0.0414, ****P <0.0001). **(C-D)** Average optical of inflammatory cytokines in corona zone. (C-D) Immunofluorescence imaging was more effective in the cell region (corona zone) (corona zone, *n*=20; non-corona zone, *n*=20; for each distance; Mann Whitney test, ****P <0.0001). **(E)** Immunofluorescence is highly expressed in the coronal region; the arrow shows the layer of coronal region. Data shown as mean ± s.e.m. Scale bar: 200 μm.
